# Who is the scientist in bio-medical research, the author or the reviewer?

**DOI:** 10.3389/fphar.2014.00050

**Published:** 2014-03-26

**Authors:** Wajahat Z. Mehal

**Affiliations:** ^1^Section of Digestive Diseases, Yale UniversityNew Haven, CT, USA; ^2^Department of Veterans Affairs Connecticut HealthcareWest Haven, CT, USA

**Keywords:** hypothesis, negative data, fibrosis, liver, author

The biology of fibrosis is an excellent example of a biological process of great clinical relevance in which many different cells and molecular pathways are interacting with each other. Many of the key cells and molecules have been identified, yet there is a lack of understanding of how the whole system can be manipulated at different stages from initial fibrogenesis, established fibrosis and during regression. When dealing with such complex systems hypothesis driven research has been the central driving force of scientific discovery. Such research begins by developing a hypothesis followed by a process of using the methods of analysis to prove its validity. In the context of biological research, “proving” is most simply explained as “testing”. The more stringent the test, the greater the likelihood that the hypothesis is sound, and represents the reality. In contrast to other powerful ideas, hypothesis driven research cannot be credited to a single event, or person. There is no Newton's *Principia*, or Darwin's *Origins*, that can be used as inspiration and as a cornerstone. The idea of hypothesis driven research coalesced around a number of trends and people. This general shift into existence also leaves it open to a drift into something else, something that should be recognized as failing the central tenant of the hypothesis driven process which is to test the hypothesis under rigorous conditions.

The reality of such a drift is most easily identified in the current process of undertaking biomedical research and then assembling, writing, and submitting an article. Although authors are intimately familiar with the basis of hypothesis driven research, what is actually done frequently resembles an attempt to highlight and showcase the hypothesis. An example is the use of *in vivo* experimental models to demonstrate the role of a molecule in a particular disease process. Subtleties of the model and the time points for analysis are often selected to provide positive data, which has silently become the surrogate goal of scientific projects. After submission of the manuscript, the interaction with reviewers often most resembles a scientific and psychological joust, with the reviewer acting as the surrogate tester for the author's hypothesis.

In many ways this is unavoidable, as the review process is a global quality control measure. Shifting the responsibility of testing a hypothesis onto the reviewer is however deeply flawed. After the structure of the project is in place, and has been executed, there is relatively little room for the reviewer to maneuver. To engineer a rigorous test of the hypothesis after the work has been conducted will require in most cases an unacceptable level of redesign and new experimental data. The limits of the current situation can be seen in the expanding pool of molecules which are shown to be absolutely vital for an experimental disease process such as fibrosis. Each report of the lack of fibrosis in the absence of a particular molecule is progress; however from a larger perspective such individual and disconnected bodies of information may obfuscate opportunities for more important insights. Are all these molecules equally important in the development of fibrosis; are there particular situations where some are more important than others?

Any workable change will have to be built into the experimental process, and not be engineered by a reviewer after completion of the project. This requires us to address the role of negative data in biological studies. Negative data is frequently seen as a failure at an experimental or conceptual level, and even as a threat to the overall research project. Should negative data be deemed a failure that could financially and intellectually bankrupt a research group? There are clearly situations where negative data is expected and is informative. A simple example is a dose response curve, where the contribution of positive and negative data in the generation of new information is in balance. This example provides a useful insight into one way by which to also regain the balance in the author-reviewer relationship for the responsibility of testing the hypothesis. For positive findings there should to be an expectation that in addition to providing evidence supporting the hypothesis the author needs to experimentally demonstrate the *limits* of the findings. With the example of *in vivo* models of fibrosis, is there a difference between the experimental and control groups at all time points (or just the one time point selected to show a difference), in many strains of mice, in multiple organs? All these questions cannot be answered for every positive finding in every paper. If however all papers are expected to provide experimental data which actively tests the limits of their findings, there will be a much greater likelihood that the findings from individual studies can be related to each other. This will rebalance our relationship to negative data which will be seen as better defining the position of the positive data in relation to the rest of the field. Most importantly it will place the author back into the role of the scientist which is a good place to be.

